# Animal models of metabolic syndrome: a review

**DOI:** 10.1186/s12986-016-0123-9

**Published:** 2016-10-04

**Authors:** Sok Kuan Wong, Kok-Yong Chin, Farihah Hj Suhaimi, Ahmad Fairus, Soelaiman Ima-Nirwana

**Affiliations:** 1 Department of Pharmacology, Faculty of Medicine, Universiti Kebangsaan Malaysia, Jalan Yaakob Latif, Bandar Tun Razak, 56000 Cheras, Kuala Lumpur Malaysia; 2 Department of Anatomy, Faculty of Medicine, Universiti Kebangsaan Malaysia, Jalan Yaakob Latif, Bandar Tun Razak, 56000 Cheras, Kuala Lumpur Malaysia

**Keywords:** Antipsychotic drugs, Carbohydrate, Fat, Fructose, Glucocorticoid, Leptin, Sucrose

## Abstract

Metabolic syndrome (MetS) consists of several medical conditions that collectively predict the risk for cardiovascular disease better than the sum of individual conditions. The risk of developing MetS in human depends on synergy of both genetic and environmental factors. Being a multifactorial condition with alarming rate of prevalence nowadays, establishment of appropriate experimental animal models mimicking the disease state in humans is crucial in order to solve the difficulties in evaluating the pathophysiology of MetS in human. This review aims to summarize the underlying mechanisms involved in the pathophysiology of dietary, genetic, and pharmacological models of MetS. Furthermore, we will discuss the usefulness, suitability, pros and cons of these animal models. Even though numerous animal models of MetS have been established, further investigations on the invention of new animal model and clarification of plausible mechanisms are still necessary to confer a better understanding to researchers on the selection of animal models for their studies.

## Background

Metabolic Syndrome (MetS) is characterized by the simultaneous occurrence of at least three of the following medical conditions, obesity, hyperglycemia, hypertension or dyslipidemia [1]. Metabolic syndrome poses a public healthcare problem worldwide owing to its increasing prevalence. Worldwide prevalence of MetS ranges from 10 to 84 % depending on age, gender, race, ethnicity and definition of MetS [2]. Approximately 20–25 % of world’s adult population is estimated to have MetS [3]. The prevalence of MetS in Malaysia was 22.9, 16.5 and 6.4 % based on the definitions by International Diabetes Federation (IDF), National Cholesterol Education Programme Adult Treatment Panel III (NCEP ATP III) and modified World Health Organization (WHO) respectively; whereby men have a higher prevalence compared to women [4].

Metabolic syndrome is a collection of various conditions, thus it does not have a single cause. Contributing factors for the features of MetS can be hereditary or environmental. Family history of type II diabetes, hypertension and insulin resistance and ethnic background are inevitable genetic factors that greatly increase the risk for developing MetS [5–8]. Furthermore, senescence is another important unalterable risk factor for MetS [9–11]. On the other hand, environmental risk factors for MetS are controllable. These include sedentary lifestyle, physical inactivity and eating habits [12]. Metabolic syndrome ultimately predisposes an individual to other medical complications. For instance, MetS causes increased risk of cardiovascular disease (CVD) [13], type II diabetes [14], non-alcoholic fatty liver disease [15], cancer (liver, pancreas, breast and bladder) [16–19], kidney and pancreatic dysfunction [20].

The deleterious effects of MetS draw research efforts in developing new interventions to reduce its burden on the healthcare system. Due to its multifactorial nature, selecting an adequate experimental model that best represents the pathophysiology of MetS in humans can be rather challenging. Rats and mice are the most common animal models used in investigating MetS. Some of the various approaches used to induce MetS in rodents include dietary manipulation, genetic modification and drugs. Previously, a review was produced by Panchal and Brown, which primarily suggested the rat model that displayed closest criteria to human MetS was induced by high-carbohydrate high-fat diet [20]. In this review, we collate and discuss the various animal models of MetS. The caveats and suitability of MetS animal models for research will also be discussed to provide the readers a comprehensive overview on the selection of the best animal models to meet their research purpose.

### Diet-induced models of MetS

Numerous dietary approaches capable to induce MetS in animals have been reported. They included the use of a single type of diet or a combination of diets, such as high-fructose, high-sucrose (Table 1), high-fat (Table 2), high-fructose/high-fat, or high-sucrose/high-fat diets (Table 3). A number of dietary studies have become the cornerstone for the investigation of MetS because diet affects whole-body metabolism and regulation through effects on hormones, glucose metabolism, and lipid metabolism pathways. The most commonly used rodent strains in diet-induced models of MetS include Sprague-Dawley rats, Wistar rats, C57BL/6 J mice and Golden Syrian Hamster [21–24]. Here, we look into how these different diets give rise to various illnesses of MetS.

**Table 1:** Effects of fructose- and sucrose-enriched diets on the development of MetS

Researchers (Year)	Types of diet	Treatment length	Strains of animal	Components of metabolic syndrome			
				Obesity	Hyperglycemia	Hypertension	Dyslipidemia
Thirunavukkarasu et al. [41]	High-fructose diet	3 weeks	Male Wistar rats	**-**	✓	✓	**-**
Sanchez-Lozada et al. [42]	High-fructose diet (60 %)	8 weeks	Male Sprague-Dawley rats	**-**	**-**	✓	✓
	Fructose drinking water (10 %)			**-**	**-**	✓	✓
Shahraki et al. [105]	High-fructose diet	8 weeks	Male Wistar rats	✗	✓	**-**	✓
Mahmoud and Elshazly [106]	Fructose drinking water (10 %)	12 weeks	Male Wistar rats	✓	✓	✓	✓
Mansour et al. [107]	High-fructose diet	16 weeks	Male Wistar albino rats	✓	✓	**-**	✓
Mamikutty et al. [108]	Fructose drinking water	8 weeks	Male Wistar rats	✓	✓	✓	✓
Di Luccia et al. [109]	High-fructose diet	8 weeks	Male Sprague-Dawley rats	✓	✓	**-**	✓
Jurgens et al. [35]	Fructose drinking water (15 %)	10 weeks	Male NMRI mice	✓	**-**	**-**	**-**
	Sucrose soft drink (10 %)			✗	**-**	**-**	**-**
	Non-caloric soft drink			✗	**-**	**-**	**-**
Oron-Herman et al. [51]	High-sucrose diet	7 weeks	Male spontaneously hypertensive rats	✗	✓	✓	✗
	High-fructose diet		Male Sprague-Dawley rats	✗	✓	✓	✓
Aguilera et al. [45]	Sucrose drinking water (30 %)	21 weeks	Male Wistar rats	✓	**-**	✓	✓
Vasanji et al. [49]	Sucrose drinking water (32 %)	10 weeks	Male Sprague-Dawley rats	**-**	✓	**-**	✓
Pang et al. [50]	High-sucrose diet	6 weeks	Male Sprague-Dawley rats	**-**	✗	✓	✓

Table represents the effects of fructose- and sucrose-enriched diets on each component of MetS. The symbol ‘✓’ and ‘✗’ indicate the presence and absence of significant effect of the sign of MetS respectively, while ‘**-**’ indicates the effects on the component not being evaluated in the study

**Table 2:** Effects of fat-enriched diet on the development of MetS

Researchers (Year)	Types of diet	Treatment length	Strains of animal	Components of metabolic syndrome			
				Obesity	Hyperglycemia	Hypertension	Dyslipidemia
Dobrian et al. [110]	High-fat diet	10 weeks	Male Sprague-Dawley rats	✓	**-**	✓	✓
Ghibaudi et al. [59]	High-fat diet	24 weeks	Male Sprague-Dawley weanling rats	✓	✓	**-**	✓
Rossmeisl et al. [111]	High-fat diet	8 weeks	Male C57BL/6 J mice	✓	✓	**-**	✓
			Male AKR/J (AKR) mice	✓	✓	**-**	✓
Gallou-Kabani et al. [112]	High-fat diet (60 %)	20 weeks	Male & female C57Bl/6 J mice	✓	✓	**-**	✓
			Male & female A/J mice	✓	✗	**-**	✓
Fraulob et al. [60]	High-fat diet	16 weeks	Male C57BL/6 mice	✓	✓	**-**	✓
Graham et al. [61]	High-fat diet	40 weeks	Male C57BL/6 mice	**-**	**-**	**-**	✓
Halade et al. [56]	High-fat diet	24 weeks	Female C57Bl/6 J mice	✓	**-**	**-**	**-**
Davidson et al. [113]	High-fat diet	24 weeks	Male Sprague-Dawley rats	✓	✓	**-**	✓
Pirih et al. [114]	High-fat diet	13 weeks	C57BL/6 mice (wild type)	**-**	✗	**-**	✓
			Hyperlipidemic (*Ldlr*−/−) mice	**-**	✓	**-**	✓
Podrini et al. [115]	High-fat diet	12 weeks	Female C57BL/6NTac mice	✓	✓	**-**	✓
Xu et al. [57]	High-fat diet	12 weeks	Male C57BL/6 mice	✓	**-**	**-**	**-**
Fujita and Maki [24]	High-fat diet	4 weeks	Male C57BL/6 J mice	✓	**-**	**-**	✓
Gancheva et al. [116]	High-fat diet	8 weeks	Male Wistar rats	✓	✓	**-**	✓
Li et al. [62]	High-fat diet	16 weeks	Male C57BL/6 mice	✓	✓	**-**	✓
Suman et al. [23]	High-fat diet	10 weeks	Male Wistar rats	✓	✓	✓	✓

Table represents the effects of fat-enriched diet on each component of MetS. The symbol ‘✓’ and ‘✗’ indicate the presence and absence of significant effect of the sign of MetS respectively, while ‘**-**’ indicates the effects on the component not being evaluated in the study

**Table 3:** Effects of different diet combinations on the development of MetS

Researchers (Year)	Types of diet	Treatment length	Strains of animal	Components of metabolic syndrome			
				Obesity	Hyperglycemia	Hypertension	Dyslipidemia
Poudyal et al. [29]	High-carbohydrate high-fat diet	16 weeks	Male Wistar rats	✓	✓	✓	✓
Panchal et al. [30]	High-carbohydrate high-fat diet	16 weeks	Male Wistar rats	✓	✓	✓	✓
Hao et al. [31]	High-carbohydrate high-fat diet	14 weeks	Male Wistar rats	✓	✓	✓	✓
Senaphan et al. [22]	High-carbohydrate high-fat diet	16 weeks	Male Sprague-Dawley rats	✗	✓	✓	✓
Dissard et al. [117]	High-fat high-fructose diet	32 weeks	Male C57BL/6 mice	✓	✓	✗	✓
Barrios-Ramos et al. [118]	Hypercholesterolemic diet & fructose drinking water	4 weeks	Male Wistar rats	✓	✓	✓	✓
Gancheva et al. [116]	High-fat high-fructose diet	8 weeks	Male Wistar rats	✓	✓	**-**	✓
Yang et al. [119]	High-fat high sucrose diet	4 weeks	Male C57BL/6 J mice	✓	✓	**-**	✓
Zhou et al. [120]	High-sucrose high-fat diet	48 weeks	Male Sprague-Dawley rats	✓	✓	**-**	✓

Table represents the effects of different diet combinations on each component of MetS. The symbol ‘✓’ and ‘✗’ indicate the presence and absence of significant effect of the sign of MetS respectively, while ‘**-**’ indicates the effects on the component not being evaluated in the study

### Carbohydrate-enriched diet

Carbohydrates can be divided into simple (e.g. monosaccharides and disaccharides) and complex (e.g. oligosaccharides and polysaccharides) forms. Carbohydrates are one of the essential nutrients acting as the main source of energy (short-term fuel) in the body because they are simpler to metabolize compared to fats. Adopting a sedentary lifestyle puts an individual into the conditions of high energy intake but low physical activity, thus increasing the tendency towards energy storage, overweight and finally obesity.

Carbohydrate metabolism begins from digestion in the small intestine to form glucose molecules, followed by absorption into the bloodstream and transportation into liver via the portal vein. When carbohydrate intake greatly exceeds daily energy requirements, blood glucose concentration will remain high and insulin is secreted by the pancreas to allow cells to uptake glucose. At this moment, the mechanisms involved in utilizing glucose are: (a) breakdown of glucose in the process of glycolysis, (b) glucose is converted to glycogen in the liver and muscles, and (c) insulin acts on adipose tissue to promote fatty acids synthesis and inhibit release of available fatty acids [25]. Prolonged excessive carbohydrate consumption causes sustained high glucose levels in the blood. Insulin is thus produced in proportion to lower the blood glucose. Therefore, high dietary carbohydrates are converted into fats for storage. Insulin sensitivity is also decreased. Substantial evidence has demonstrated a strong association between high carbohydrate intake and insulin resistance [26–28].

Information on the metabolic impact of carbohydrate on animal models of MetS is absent. Most of the diet regimens were designed with the combination of high-carbohydrate and high-fat. Two studies tracing metabolic changes in rats fed with high-carbohydrate high-fat diet are available. These studies adopted a high-carbohydrate high-fat diet (consist of 39.5 % sweetened condensed milk, 20 % beef tallow, 17.5 % fructose, 15.5 % powdered rat food, 2.5 % salt mixture, 5 % water) to induce MetS in an animal model. The researchers claimed it mimics more closely the human disease state compared to other methods of inducing MetS [29–31]. Test animals developed hypertension, impaired glucose tolerance, increased abdominal fat deposition, increased abdominal circumference, and altered lipid profile after 16 weeks on this diet. Another study by Senaphan and co-workers reported that high-carbohydrate high-fat diet with some modifications (35 % sweetened condensed milk, 20 % pork tallow, 17.5 % fructose, 20 % powdered rat food, 2.5 % salt mixture, 5 % water) provided similar outcomes as the previous study [22].

Ironically, the combination of high-carbohydrate with high-fiber was reported to confer hypolipidemic and hypoglycemic effects as evidenced in human studies. In a clinical study, high-carbohydrate high-fiber diet was suggested as dietary therapy in diabetic patients because this diet was capable of reducing postprandial plasma glucose, insulin response, cholesterol, and triglycerides levels [32]. Hence, the composition and combination of high-carbohydrate diet are important factors that must be taken into consideration for the induction of MetS.

### Fructose-enriched diet

Fructose, commonly known as the fruit sugar, is one of the monosaccharides along with glucose and galactose. Nowadays, fructose is often used as a taste enhancer to make food more appetizing and tempting. There is no biological need for dietary fructose; it is only an intermediary molecule during glucose metabolism. The circulating concentration of fructose (~0.01 mmol/L) in peripheral blood is very low compared to glucose (~5.5 mmol/L) [33]. Interestingly, a small quantity of fructose produces a lower glycemic response to substitute sucrose and starch in the diet in diabetic patients [34]. Unfortunately, intake of fructose is excessive nowadays due to the consumption of artificially sweetened beverages and food.

Theoretically, a large influx of fructose into the liver causes accumulation of triglycerides and cholesterol because of its lipogenic (fat-producing) properties, subsequently leading to reduced insulin sensitivity, insulin resistance and glucose intolerance [35, 36]. Fructose consumption resulted in massive fructose uptake by the liver. Fructose is converted to fructose-1-phosphate, a reaction catalyzed by the enzyme phosphofructokinase in the presence of ATP. It is followed by the cleavage of fructose-1-phosphate into glyceraldehyde and dihydroxyacetone phosphate without the conversion of glucose to fructose-1,6-bisphosphate, an initial regulatory step of glycolysis [37]. Phosphofructokinase is a negative regulator for glucose metabolism, allowing fructose to enter into the glycolysis pathway continuously. Fructose-1,6-bisphosphate is then converted to pyruvate through the process of glycolysis. At this juncture, fructose is involved in several simultaneous processes: (a) a portion of the fructose is converted into lactate from pyruvate, (b) another portion produces triose-phosphate which readily converts to glucose or glycogen via gluconeogenesis, (c) carbons derived from the fructose can be converted into fatty acids, and (d) inhibition of hepatic lipid oxidation by fructose favours very low density lipoproteins (VLDL)-triglyceride synthesis and fatty acid re-esterification [38]. As a result, this refined carbohydrate is rapidly absorbed and readily metabolized by liver to produce glucose, glycogen, pyruvate, lactate, glycerol, and acyl-glycerol molecules.

Knowledge on fructose metabolism revealed the superiority of fructose-feeding for the induction of MetS in animal models in comparison with glucose or starch. Previous research indicated that glucose or starch-feeding is not as effective as fructose-feeding in inducing MetS [39]. In addition, mice fed with fructose gained more weight compared to mice fed with the same calories using starch [35]. The correlation between chronic high intake of dietary fructose with increased energy intake, body weight, adiposity, hypertriglyceridemia, hyperlipidemia, hypertension, glucose intolerance and decreased insulin sensitivity in laboratory animal, all leading to MetS, is indisputable [39, 40]. An animal study conducted by Thirunavukkarasu et al. [41] showed that increased blood pressure, glucose intolerance, and decreased insulin sensitivity were detected in rats fed with a fructose-enriched diet containing >60 % of total calories. Another study performed by Sanchez-Lozada et al. [42] reported that 10 % of fructose in drinking water resulted in the same effects as high dose of fructose (60 % in diet) in inducing hypertension and hyperlipidemia in male Sprague-Dawley rats, but they were less severe compared to high dose of fructose.

To sum up, fructose behaves more like a fat instead of a carbohydrate in both humans and animals. A low dose of fructose in drinking water (10 %) is sufficient to induce MetS in animals.

### Sucrose-enriched diet

Sucrose, or table sugar, is a disaccharide found in cane or beet sugar. It consists of one fructose molecule and one glucose molecule. Sucrose has the same role as fructose to make food more palatable. When sucrose is consumed, it is cleaved into its constituents, i.e. glucose and fructose by the enzyme sucrase [43]. Both molecules are then taken up by their specific transport mechanisms. As outlined earlier, glucose uptake in glucose metabolism is negatively regulated by phosphofructokinase, leading to the continuous entry of fructose into the glycolytic pathway. Excess fructose will be converted into fat in the liver as fructose is a better substrate for fatty acid synthesis compared to glucose [44]. Thus, fructose is the main active ingredient contributing to the development of MetS in animals after sucrose consumption.

An animal study showed that administration of 30 % sucrose in drinking water led to the development of MetS in male Wistar rats with increased body weight, systolic blood pressure, insulin, triacylglycerol, total cholesterol, low density lipoproteins (LDL) cholesterol, and free fatty acids [45]. Besides, high sucrose supplementation is widely used for induction of whole body insulin resistance in rats, whereby high levels of plasma insulin was detected [45–48]. Concomitantly, animals treated with 32 % sucrose in drinking water exhibited hyperglycemia, hypertriglyceridemia, hypercholesterolemia, and increased body weight [49]. Another study by Pang et al. [50] reported that rats responded to sucrose supplementation (77 %) by a significant elevation in systolic blood pressure, plasma insulin, and triglycerides.

However, Kasim-Karakas et al. [21] revealed that only fructose-feeding increased fasting non-esterified fatty acids and triglycerides levels in the plasma and liver of Golden Syrian hamsters. However the increment was not found in sucrose-fed hamsters. Moreover, impaired glucose tolerance, significant increase of body weight and body fat were only detected in fructose-fed (15 %) rats, but not in other groups fed with a soft drink (10 % sucrose) and a diet soft drink (without calories) [35]. Fructose and sucrose supplementation also invoked distinct responses in two different animal models, i.e. Sprague-Dawley and spontaneous hypertensive rats, which represented environmentally and genetically acquired MetS respectively. Fructose enrichment in Sprague-Dawley rats caused hyperinsulinemia, hypertriglyceridemia, hypercholesterolemia, hypertension, and insulin resistance. Meanwhile, sucrose enrichment in spontaneous hypertensive rats only increased blood pressure and worsened insulin resistance [51].

These paradoxical outcomes accumulated from previous studies implied that high content of sucrose will ensure the success of MetS development in animal models. However, fructose appeared to be more superior than an equivalent amount of sucrose in inducing MetS because fructose exists as a free molecule while sucrose contains only 50 % fructose and 50 % glucose.

### Fat-enriched diet

Fats are one of the three main macronutrients and are the most calorically dense macronutrient [52]. Fats, also known as triglycerides, are composed of esters of three fatty acid chains and glycerol. Lipid metabolism begins with the process of lipolysis. Plenty of glycerol and fatty acids diffuse freely into the bloodstream. Plasma free fatty acids are major substrates for hepatic VLDL-triglycerides production [53]. Approximately 70 % of released free fatty acids will be re-esterified (lipogenesis) to form triglycerides [54]. The rate of re-esterification is dependent on the rate of glycerol-3-phosphate production through glycolysis and the rate of fatty acid release from adipocytes [25]. The coupled actions of free and re-esterified fatty acids (triglycerides) form VLDL, which assists fats to circulate in the water-based solution of the bloodstream.

Many researchers have employed different types of high-fat diets that vary between 20 and 60 % of total energy. The source of the fat component may be either plant-derived oils (e.g. corn, safflower or olive oil) or animal-derived fats (e.g. beef tallow and lard) [55]. High-fat diets have been extensively used to induce MetS in experimental animals. More specifically, high-fat diets have been widely used to induce obesity in animals [56, 57]. Studies have also indicated that high-fat diet is effective in promoting hyperglycemia, insulin resistance, dyslipidemia and increased free fatty acids in the blood, either independently or concurrently [58].

A comprehensive study by Ghibaudi et al. [59] aimed to assess the chronic effect of dietary fats with different fat content (10, 32 and 45 %) on body adiposity and metabolism in rats. The findings demonstrated that energy intake, weight gain, fat mass, plasma glucose, cholesterol, triglycerides, free fatty acids, leptin, and insulin levels increased dose-dependently with increased dietary fat. Apart from that, mice fed with high-fat (60 %) diet exhibited greatly increased body mass, total fat pads, plasma triglyceride, high density lipoproteins (HDL) cholesterol, and LDL cholesterol levels [60]. Another animal model fed with high-fat diet displayed elevation of total cholesterol, LDL cholesterol, and unesterified cholesterol [61]. Later investigation has found that high-fat intake augmented body weight, total cholesterol, and leptin levels in male C57BL/6 J mice [24]. Another recent study indicated that mice fed with high-fat diet had increased body weight, plasma lipids, plasma insulin, and insulin resistance compared to mice fed with standard chow [62]. To conclude, the increased formation of VLDL helps to distribute assembled triglycerides synthesized by the liver resulting from overconsumption of high-fat diet. A high level of VLDL cholesterol can cause obesity, dyslipidemia and the build-up of cholesterol in arteries. The accumulation of triglycerides in the liver can cause insulin resistance.

**Fig. 1:**
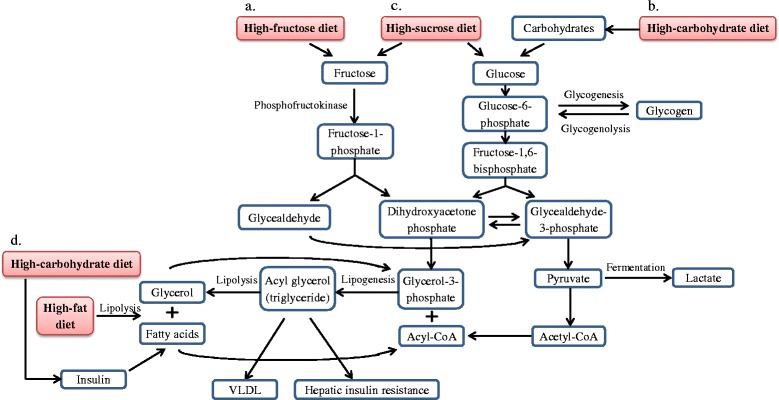
Summary of the effects of different diets on whole body metabolism. **a** High-fructose diet intake interferes glycolytic pathway by bypassing the rate-controlling step, the conversion of glucose-6-phosphate into fructose-1,6-bisphosphate. Phosphofructokinase acts as a negative regulator for glucose metabolism and allows fructose to enter the glycolytic pathway continuously to produce pyruvate, lactate, glycerol and acyl-glycerol. **b** When plenty of glucose is available during high dietary carbohydrate, glucose utilizing pathways are initiated: breakdown of glucose by glycolysis, conversion of glucose into glycogen via glycogenesis, and production of insulin which acts on adipose tissue to promote fatty acids synthesis. **c** Consumption of high-sucrose diet: sucrose separates into fructose and glucose molecules and enters their specific mechanisms as stated earlier. **d** Fats undergo lipolysis, glycerol and fatty acids are released into the blood. However, fatty acids released during lipolysis are re-esterified to form triglyceride. Overproduction of triglyceride through excessive intake of various nutrients is likely to cause accumulation of triglyceride in the liver, which will further lead to hepatic insulin resistance (reduced insulin sensitivity)

A summary on the effects of different nutrients on whole body metabolism has been illustrated (Fig. 1). The lipotoxicity hypothesis (overproduction and accumulation of triglycerides in the non-adipose tissue such as liver, muscle, and pancreas) is the common criteria seen in the effect of different diets in the development of MetS. It is alarming that MetS can be contributed by these seemingly harmless essential nutrients.

### Genetic models of MetS

In addition to diet-induced MetS animal model, genetic animal models are imperative in order to investigate the pathogenesis of MetS caused by genetic factors. These genetic models of MetS are time-saving because the duration for the development of MetS is significantly shortened compared to diet-induced MetS.

Originally, leptin- or leptin receptor-deficient rodent models are used as genetically obese and diabetic experimental models. Numerous animal models are developed, such as leptin-deficient (*ob/ob*) mice, leptin receptor-deficient (*db/db*) mice, Zucker fatty (ZF) rats, Zucker diabetic fatty (ZDF) rats, DahlS.Z-*Lepr* ^*fa*^ */Lepr* ^*fa*^ (DS/obese) rats, Goto-Kakizaki (GK) rats, obese spontaneous hypertensive rat (Koletsky rat), and the POUND mice™. Leptin, serving as an anti-obesity hormone by binding to leptin receptor, is secreted by mature adipocytes in proportion with the size of fat depots [63]. Circulating leptin is taken up into the hypothalamus to decrease food intake and eating appetite to increase energy expenditure via several signaling pathways. Thus, the occurrence of obesity in these models is basically owing to the abnormalities in leptin signaling, which result in hyperphagia (great desire on food), uncontrolled appetite, and reduced energy expenditure [64].

Leptin-deficient (*ob/ob*) and leptin receptor-deficient (*db/db*) mice are the models of single autosomal recessive mutation on leptin gene (chromosome 6) and leptin receptor gene (chromosome 4) respectively. Leptin-deficient (*ob/ob*) mice develop obesity, hyperinsulinemia and hyperglycemia with the absence of hypertension and dyslipidemia. Both hypertension and dyslipidemia did not develop even after 38 weeks of age [65]. Whereas leptin receptor-deficient (*db/db*) mice develop obesity, hyperglycemia, and dyslipidemia without hypertension [66, 67]. Hence, both of these animal models are excellent models for obesity and type II diabetes, but not for MetS. Zucker fatty rat, also known as leptin receptor-deficient obese rat, carries a missense mutation in the leptin receptor gene with homozygous *fa* allele, hallmarked by an increased circulating leptin level [68]. Obesity developed in these rats as early as between 3 to 5 weeks of life [69]. Instead of being genetically obese, ZF rats demonstrated hyperinsulinemia, insulin resistance, mild glucose intolerance, dyslipidemia, and hypertension [70, 71]. A variant of Zucker rat, known as Zucker Diabetic Fatty rat, is a selective inbred rat strain derived from ZF rat with high glucose levels [71]. Zucker Diabetic Fatty rats display hyperphagia caused by a non-functioning leptin receptor [72]. Moreover, ZDF rats recapitulate several phenotypes of type II diabetes (impaired glucose metabolism, hyperglycemia, and hyperinsulinemia) resulting from the defects of GLUT-2 and GLUT-4 transporter. Long-term severe diabetes leads to mild cardiac diastolic dysfunction in ZDF rats [73]. Hattori et al. [74] introduced a new animal model of MetS, namely DahlS.Z-*Lepr* ^*fa*^ */Lepr* ^*fa*^ (DS/obese) rat strain, which was established from a cross between Dahl salt-sensitive rats and ZF rats. Higher systolic blood pressure, body weight, visceral fat mass, subcutaneous fat mass, and ratio of LDL cholesterol to HDL cholesterol levels were detected in DS/obese rats compared to DahlS.Z-*Lepr* ^*+*^ */Lepr* ^*+*^ (DS/lean) rats fed on a normal diet, whereas fasting serum glucose concentration remained unchanged. After that, Murase et al. [75] further confirmed this strain of rat as a MetS animal model because female DS/obese rats developed elevated systolic blood pressure, body weight, insulin, triglycerides, LDL:HDL cholesterol ratio, visceral and subcutaneous fat mass.

Goto-Kakizaki (GK) rat, a leptin resistant animal model, is considered as one of the best non-obese inbred model of type II diabetes [76]. They spontaneously develop hyperleptinemia, hyperphagia, hyperglycemia, decreased β-cell function, increased gluconeogenesis, and accumulation of visceral fat [77, 78]. Goto-Kakizaki rat was established through repetitive selective breeding of Wistar rats with glucose intolerance over several generations [79, 80]. In light of the difficulties to access human pancreatic islet defect, this specific animal model representing human type II diabetes provide an opportunity to study the disease intensively. However, GK rats only act as genetic model representing certain aspects of MetS thus not a suitable animal model to represent MetS.

Spontaneous hypertensive rat (SHR) was generated from outbreed between Wistar Kyoto male rats with noticeable elevated blood pressure and females with slight elevation of blood pressure, followed by selective inbreed of the offspring with highest blood pressure [81]. The SHR is used as an experimental model for genetically induced hypertension. A study by Potenza et al. [82] demonstrated that 12-week-old SHRs were hypertensive, hyperinsulinemic, and insulin resistant compared to Wistar-Kyoto rats. Spontaneously hypertensive rats generally do not develop hypercholesterolemia and hyperlipidemia unless they are put on a special diet regimen, such as high-cholesterol or high-fructose high-fat diet [83, 84]. Modification of SHR, known as obese SHR or Koletsky rat, was obtained by crossing a female SHR with a normotensive Sprague-Dawley male. Koletsky rats carry a nonsense mutation in the leptin receptor and possess interesting phenotypes, including obesity at 5 weeks of age, hypertriglyceridemia even with standard diet, hyperinsulinemia with normal blood glucose, and severe hypertension at 3 months of age [69]. Koletsky rats have been suggested as a more appropriate animal model for MetS compared to SHRs. The POUND mouse (C57BL/6NCrl-*Lepr* ^*db-lb*^/Crl) was established in the last decade as another model fulfilling all the MetS criteria in a single animal. The animals were fed with Purina Diet *ad libitum* and showed obesity at 1 month of age, hyperinsulinemia and hyperglycemia at 18 weeks of age, increased leptin levels at 17–18 weeks of age, as well as increased cholesterol levels at 14 weeks of age [85].

Amongst all these leptin- and leptin receptor-related rodent models, ZF rats, ZDF rats, DS/obese rats, Koletsky rats and POUND mice are suitable models of MetS because these rats display all the conditions of MetS (Table 4). Genetic models are beneficial in elucidating the plausible molecular mechanisms involved in the development of certain disease states. However, there were only nine mutations have been identified in the leptin gene in 2014 and mutations were more prevalent in consanguineous marriages [86]. Thus mutations of leptin or leptin receptor rarely occur in humans, implying that they do not actually resemble the human disease state in real life.

**Table 4:** Metabolic changes in genetic models of MetS

Strain	Model	Mutation/deficiency	Abnormalities/metabolic changes	References
Leptin-deficient (*ob/ob*) mice	Obesity, type II diabetes	Autosomal recessive mutation on leptin gene (chromosome 6)	(a) Obese & increased body weight (Age: 4 weeks) (b) Hyperinsulinemia & hyperglycemia (Age: 4 weeks) (c) Impaired glucose tolerance (Age: 12 weeks) (d) Reduced blood pressure (e) Does not develop dyslipidemia	[65, 121, 122]
Leptin receptor-deficient (*db/db*) mice	Obesity, type II diabetes	Autosomal recessive mutation on leptin receptor gene (chromosome 4)	(a) Obese & increased body weight (Age: 6 weeks) (b) High fasting blood glucose (Age: 8 weeks) (c) Hyperinsulinemia & impaired glucose tolerance (Age: 12 weeks) (d) Unchanged blood pressure (e) Increased triglycerides, total cholesterol, LDL cholesterol, and free fatty acid (Age: 13 weeks)	[66, 67]
Zucker fatty (ZF) rat	Metabolic syndrome	Missense mutation on leptin receptor gene	(a) Obese (Age: 3-5 weeks) (b) Hyperinsulinemia, insulin resistance (Age: 3 weeks) (c) Hypertension (Age: 12 weeks) (d) Hypercholesterolemia, hypertriglyceridemia	[71]
Zucker Diabetic Fatty (ZDF) rat	Metabolic syndrome	Non-functional leptin receptor	(a) Obese (Age: 3–5 weeks) (b) Hyperinsulinemia, insulin resistance, hyperglycemia (Age: 13–15 weeks) (c) Mild hypertension (Age: 12–14 weeks) (d) Hypercholesterolemia, hypertriglyceridemia (Age: 20 weeks)	[123]
DahlS.Z-*Lepr* ^*fa*^ */Lepr* ^*fa*^(DS/obese) rat	Metabolic syndrome	-	(a) Obese (Age: 18 weeks) (b) Hyperinsulinemia (Age: 18 weeks) (c) Unchanged serum glucose concentration (d) Hypertension (Age: 11–12 weeks) (e) Hypercholesterolemia & hypertriglyceridemia (Age: 18 weeks)	[74, 75]
Goto-Kakizaki (GK) rat	Type II diabetes	Leptin resistance	(a) Non-obese (b) Hyperinsulinemia, insulin resistance & mild hyperglycemia (Age: 4 weeks) (c) Hyperlipidemia (Age: 8 weeks) (d) Unchanged blood pressure (Age: 14 months)	[76, 78, 124, 125]
Spontaneous Hypertensive rat	Hypertension	-	(a) Hyperinsulinemia, insulin resistance (Age: 12 weeks) (b) Severe hypertension (Age: 4 weeks)	[82, 126]
Obese Spontaneous Hypertensive rat (Koletsky rat)	Metabolic syndrome	Nonsense mutation of leptin receptor	(a) Obese, increased abdominal fat (Age: 5 weeks) (b) Hyperinsulinemia, insulin resistance (Age: 16–18 weeks) (c) Normal fasting blood glucose (d) Severe hypertension (Age: 12 weeks) (e) Hyperlipidemia (Age: 16–18 weeks)	[69, 127]
The POUND mouse™	Pre-diabetes/metabolic syndrome	Mutation in leptin receptor (deletion of axon 2 on chromosome 4)	(a) Obese (Age: 4 weeks) (b) Hyperinsulinemia, hyperglycemia (Age: 18 weeks) (c) Increased leptin levels (Age: 17–18 weeks), (d) Hypercholesterolemia (Age: 14 weeks)	[85, 128]

### Drug/chemically-induced model of MetS

#### Glucocorticoid-induced MetS

Endogenous glucocorticoids are naturally occurring stress hormones secreted by the adrenal glands. Glucocorticoids bind to its receptors (glucocorticoid and mineralocorticoid receptors) to exert their effects on different tissues [87]. Apart from that, exogenous glucocorticoids are used as medicine to treat a wide range of human diseases, such as autoimmune disease and cancer. It is also used to prevent rejection in organ transplantation. However, glucocorticoid treatment brings about undesirable side effects such as body weight gain, glucose intolerance, impaired calcium homeostasis, osteoporosis, cataracts and central nervous system effects [88]. Both endogenous and exogenous glucocorticoids have been used to develop MetS in animal models [89].

Glucocorticoids cause MetS by acting directly on different tissues and organs (e.g. fat, liver, muscles, and kidneys) via several mechanisms: (1) glucocorticoids stimulate the differentiation of pre-adipocytes into mature adipocytes; (2) glucocorticoids increase lipolysis to release free fatty acids; (3) glucocorticoids increase proteolysis in muscle to increase free amino acids. Amino acid-induced mammalian target of rapamycin complex-1 (mTORC1) activation causes phosphorylation of insulin receptor substrate-1 (IRS-1), leading to the occurrence of insulin resistance; (4) glucocorticoids promote gluconeogenesis in liver and cause hyperglycemia; and (5) non-specific binding of glucocorticoids to its receptor in the kidneys causes an increase in sodium retention, potassium excretion, water retention, and plasma volume concomitantly with elevation of blood pressure [87, 88, 90].

Using laboratory animals, glucocorticoid-induced MetS has been done through various approaches, such as feeding [87, 91], daily intraperitoneal injections [92], or surgically implanted glucocorticoid pellets [93, 94]. All these different routes of administration of glucocorticoids resulted in almost similar outcomes. Mounting levels of corticosterone enhanced food intake, weight gain, abdominal fat accumulation, severe fasting hyperglycemia, insulin resistance, impaired glucose tolerance, hypertension, dyslipidemia, as well as deposition of lipids in visceral adipose, hepatic tissue and skeletal muscle in animals. Meanwhile, the removal of corticosterone reversed all these adverse conditions.

#### Antipsychotic-induced MetS

Antipsychotic drugs are medications used to treat neuropsychiatric disorders, for examples, schizophrenia, depression, and bipolar disorder [95]. Antipsychotic drugs have been associated with a high incidence of MetS, evidenced by body weight gain, increased visceral fat, impaired glucose tolerance, and insulin resistance in animal studies [96, 97]. However, the exact underlying mechanism involved in antipsychotic-induced MetS still remains an enigma. The proposed mechanism available currently is that the weight gain caused by antipsychotic treatment contributes to the development of diabetes and dyslipidemia [98]. Latest evidence demonstrated that administration of the second generation antipsychotic, olanzapine, via intraperitoneal injection or oral gavage interacted with gut microbiota and caused body weight gain, increased plasma free fatty acids, infiltration of macrophages in adipose tissue, and deposition of visceral fat in both rat and mouse models [97, 99]. Since antipsychotic drugs are important as treatment for psychiatric diseases, ongoing research is necessary to elucidate the plausible mechanisms involved in antipsychotic-induced MetS so that this side-effect can be avoided. The comparison between various types of MetS animal model has been summarized (Table 5).

**Table 5:** Overall merits and caveats of various types of MetS animal model

Diet-induced model of MetS Examples: Induction by fructose drinking water, high fat diet, and high-carbohydrate high-fat diet	
Pros	Cons
➢ Suitable for the investigations of non-genetic lifestyle-dependent MetS in humans ➢ Inexpensive (dependent on the kind of diet)	➢ Delayed onset of MetS ➢ A lengthy duration of diet regimen (usually takes up to 16 weeks)
Genetic model of MetS Examples: ZF rat, ZDF rat, DS/obese rat, Koletsky rat, POUND mouse	
Pros	Cons
➢ Severe and spontaneous occurring MetS	➢ Do not resemble the criteria of MetS in humans with intact leptin receptor gene ➢ Mutation in leptin or leptin receptor gene rarely occur in humans ➢ Costly ➢ Mutations/deficiencies in animals are not easily manipulated
Drug/chemical-induced model of MetS Example: Induction by glucocorticoids and antipsychotic drugs	
Pros	Cons
➢ Suitable for the investigations of drug-related MetS in human ➢ Inexpensive	➢ Delayed onset of MetS

#### Other animal models of MetS

Other animal models of MetS are available despite those typical laboratory rodent models, such as the use of guinea pig, swine, Nile rat, and Sand rat. A male Hartley guinea pig model of MetS was successfully developed by exposure to high-fat, high-sucrose or high-fat high-fructose diet for 150 days [100, 101]. Additionally, Ossabaw swine model of MetS was developed after fed with high-fat, high-cholesterol atherogenic diet, evidenced by obesity, elevated arterial pressure, glucose intolerance, and hyperinsulinemia [102]. Nile rat (*Arvicanthis niloticus*) was introduced as a novel model of MetS that experiences onset of hyperglycemia, hypertension, dyslipidemia, and abdominal fat accumulation by age of one when rats were given laboratory chow diet [103]. Sand rat (*Psammomys obesus*), found mostly in North Africa, spontaneously develops obesity and diabetes under laboratory diets [104]. These MetS features have not been observed among the wild type of Nile and Sand rats.

## Conclusions

In conclusion, the advantage of using animal models to study MetS is the ability to monitor histological, functional, biochemical, and morphological changes of MetS, which is difficult to conduct in humans. Subsequent studies are encouraged using combination or modification of existing established methods in order to successfully develop an animal model of MetS with the desired metabolic changes. Apart from pathophysiological similarity with human MetS, an excellent animal model should also be reproducible, simple, reliable, and affordable with minimal disadvantages.

## Abbreviations

CVD: Cardiovascular disease
*db/db* mice: leptin receptor-deficient mice
DS/lean rats: DahlS.Z-*Lepr* ^*+*^ */Lepr* ^*+*^ rats
DS/obese rats: DahlS.Z-*Lepr* ^*fa*^ */Lepr* ^*fa*^ rats
GLUT-2: glucose transporter-2
GLUT-4: glucose transporter-4
GK rats: Goto-Kakizaki rats
HDL: High density lipoproteins
IDF: International Diabetes Federation
IRS-1: Insulin receptor substrate-1
LDL: Low density lipoproteins
MetS: Metabolic syndrome
mTORC1: Mammalian target of rapamycin complex-1
NCEP ATP III: National Cholesterol Education Programme Adult Treatment Panel III
*ob/ob* mice: Leptin-deficient mice
SHR: Spontaneous hypertensive rat
VLDL: Very low density lipoproteins
WHO: World Health Organization
ZDF rats: Zucker diabetic fatty rats
ZF rats: Zucker fatty rats
